# Collective Motion and Programmable Self-Organization of Rotating Active Particles Manipulated by Magnetic Fields

**DOI:** 10.3390/mi17091068

**Published:** 2026-09-09

**Authors:** Kailai Wang, Jiawei Li, Leilei Wang, Shishuang Zhang, Xu Zheng, Qilong Zheng, Haihang Cui

**Affiliations:** 1 State Key Laboratory of Nonlinear Mechanics, Institute of Mechanics, Chinese Academy of Sciences, Beijing 100190, China; wangkailai@xauat.edu.cn (K.W.); zhengxu@lnm.imech.ac.cn (X.Z.); 2 School of Building Services Science and Engineering, Xi’an University of Architecture and Technology, Xi’an 710055, China; 13939810562@163.com; 3 Xi’an Modern Chemistry Research Institute, Xi’an 710065, China; ljw3656@163.com (J.L.); zhenglong2577@163.com (Q.Z.)

**Keywords:** active particle, rotating magnetic microparticles, hydrodynamic coupling, phase separation, finite Reynolds number flow

## Abstract

Rotating magnetic microparticles are classic active matter systems dominated by competing magnetic dipolar attraction and spin-induced hydrodynamic repulsion, whose collective behaviors under programmable magnetic field remain insufficiently characterized. This work builds a two-dimensional orthogonal Helmholtz coil experimental setup to investigate the collective motion and self-organization of magnetic microparticles. For single-component assemblies, the hexatic order parameter varies non-monotonically with driving frequency and particle area fraction; an intermediate frequency range (60–80 Hz) yields optimal hexagonally ordered structures, and a full phase diagram covering clustered, ordered and disordered states is established. Binary mixtures of 200 μm and 300 μm particles display hydrodynamic driven size segregation, with the segregation parameter peaking uniformly at 60 Hz. By applying programmable Lissajous-type magnetic fields with mismatched orthogonal frequencies, we achieve tunable elliptical particle trajectories and controlled splitting of particle clusters. This study reveals the coupling mechanism between magnetic and finite Reynolds number hydrodynamic interactions and proposes a programmable method to dynamically reconfigure active microparticle swarms.

## 1. Introduction

Active matter refers to a class of non-equilibrium systems composed of units that consume energy and convert it into mechanical motion, spanning a vast range of scales in nature from molecular motors and cells to biological collectives [1,2,3]. The defining feature of active matter lies in its self-propulsion and energy-dissipating nature. Unlike equilibrium systems governed by free-energy minimization, the interactions between active matter units are fundamentally non-conservative, and the system’s evolution cannot be described by the principles of equilibrium statistical mechanics but instead requires a non-equilibrium framework [4,5,6,7]. In recent years, the construction of artificial active matter systems by driving colloidal particles or microrobots through external fields has emerged as a powerful experimental platform for understanding the emergence of collective behaviors in living or other complex systems [8]. Among these, rotating active particles, also known as spinners, has attracted broad interest from both the physics and engineering communities, owing to their rich collective dynamics and unique hydrodynamic coupling effects [9].

Due to the moderate size, excellent visualizability, and high degree of parameter tunability, rotating active particle systems controlled by magnetic field has been widely used as highly controllable platforms to study the emergence of collective behaviors [10,11] and non-equilibrium phase transitions in active matter [12,13], where particle–particle interactions are governed by hydrodynamic coupling, magnetic dipole forces, and chirality [14,15]. Magnetic actuation provides a noncontact and spatially temporally precise means of control, enabling independent manipulation of particle rotation frequency, phase, and even chirality, thus offering an ideal experimental condition for systematic investigation of collective rotational motion [9,16,17]. Early studies demonstrated that at thermal energy scales particles can form rotating dimers at low frequencies via magnetic dipole–dipole interactions, and transition to independently rotating states at high frequencies through hydrodynamic coupling [14], revealing a frequency-dependent force and torque transmission mechanism. Intriguingly, rotating active particles can self-organize into diverse ordered structures through purely hydrodynamic interactions, without any direct attractive forces or thermal fluctuations, including spinodal phase separation of co-rotating rotors [15,18], flocking of ferromagnetic colloids via spontaneous symmetry breaking, and the formation of dynamic self-assembled patterns at interfaces [19]. The recent literature focused on micro/nanorobot collectives [16,20,21] for achieving reconfigurable, adaptive, and multifunctional collective behaviors. Critical mechanisms contain how chirality and odd viscosity arrest phase separation coarsening [22], how inertial effects and lift forces drive cavitation instability and two-phase crystallization in dense spinner monolayers [23,24], and how heterogeneity and field programmability can be exploited to achieve functionally diverse self-organization in micro/nanorobot collectives [10,21,25].

Despite significant progress in identifying such emergent phenomena, from purely hydrodynamic ordering to spinodal and arrested phase separation, existing studies remain largely confined to homogeneous rotating particle populations at specific Reynolds number (*Re*) regimes, leaving the systematic mapping of collective phase diagrams across inertial-to-viscous transitions, the competition between magnetic and hydrodynamic interactions, and the role of multi-parameter heterogeneity unexplored. Therefore, we established an experimental system to study the collective motion of magnetic rotating particle systems. We first systematically investigate the effects of rotation frequency and area fraction on the hexatic order of particle collectives and present the corresponding phase diagram. We then characterize the feature phase separation structures in a binary size-heterogeneous system. Addressing the gap in prior studies that largely overlooked the influence of magnetic field signal modulation, we reveal the formation and control of non-circular rotational patterns through programmed rotating magnetic field waveforms, indicating the possibility of a Lissajous figure-like manipulation strategy.

## 2. Materials and Methods

**Figure 1:**
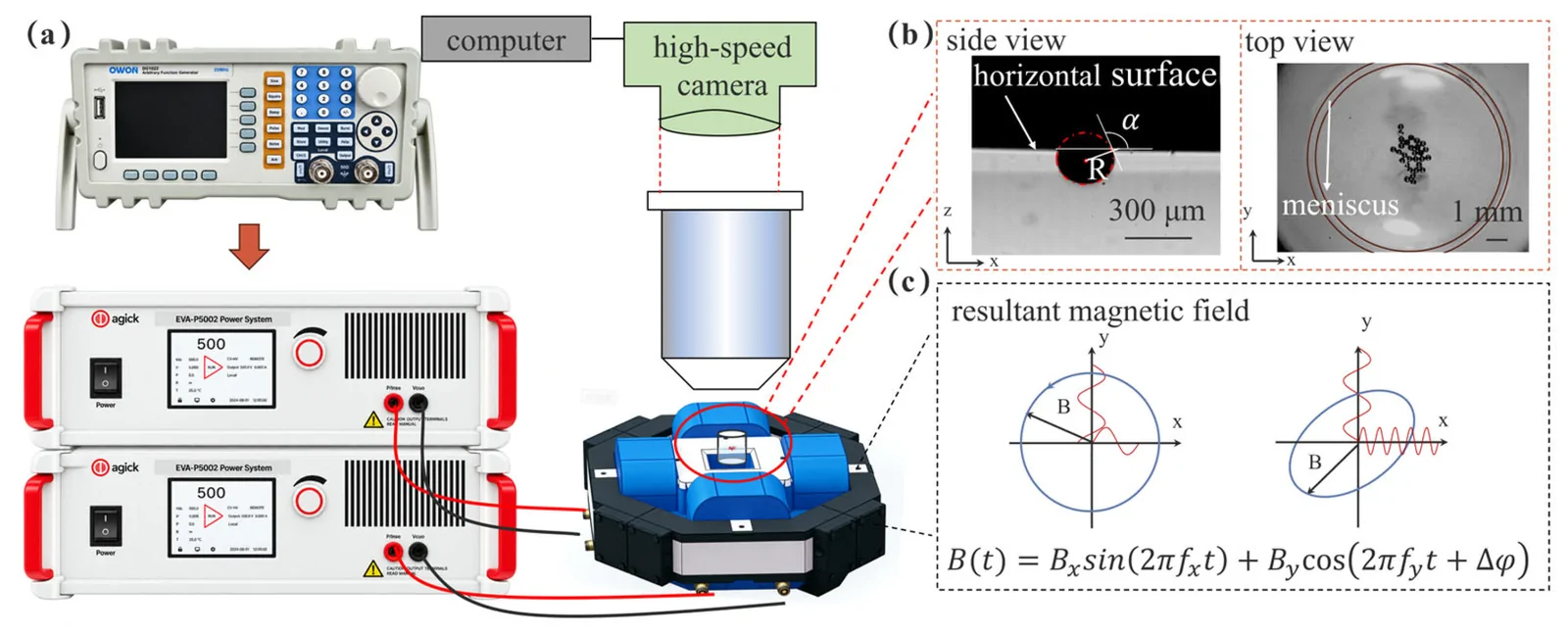
Experimental system and magnetic field-regulated collective behaviors of magnetic microparticles. (**a**) Schematic of the experimental setup and the magnetic actuation system. (**b**) Experimental observation of magnetic microparticles at the air–liquid interface. The red dashed line outlines the particle boundary and the red solid lines indicate the outer and inner contours of the liquid meniscus, respectively. (**c**) The programmable rotational magnetic field is established by the superposition of sine/cosine waves along the *x* and *y* axes.

The experimental system mainly consisted of a magnetic actuation system, a two-dimensional orthogonal Helmholtz coil setup, and a microscopic imaging system. The magnetic actuation system was composed of a signal generator and power amplifiers. Programmable electrical signals generated by the signal generator were amplified and then applied to two pairs of orthogonal Helmholtz coils, producing a two-dimensional uniform magnetic field within a circular region with a radius of approximately 1.0 cm at the center of the coil system. The magnetic field uniformity within this region was greater than 90%, as further characterized with numerical simulation [26] in Figure S1 of the Supplementary Information. This configuration enabled precise control of the rotation, spinning, and collective motion of magnetic particles at the air–liquid interface. The microscopic imaging system consisted of a high-speed camera (Phantom V7.3) and a stereomicroscope (Leica Z16APO), as shown in Figure 1a, and was used to record the dynamic motion of particles in the target region for subsequent kinematic analysis and structural characterization.

The spherical magnetic microparticles used in this study were prepared by depositing a Ni thin film (with thickness of approximately 1 μm) onto polystyrene (PS) microspheres via magnetron sputtering, thereby imparting magnetic responsiveness to the particles. Single-component experiments were conducted using 300 μm magnetic microparticles, and binary experiments were performed using two types of magnetic microparticles with diameters of 200 μm and 300 μm. The equilibrium positions of the two types of particles at the air–liquid interface were characterized by the angles α and *β* between the tangential direction of the particle and the horizontal plane, both of which were approximately 111.8°. As shown in Figure 1c, this study mainly investigated the structural evolution of 300 μm microparticle assemblies, the phase separation behavior of 200 μm and 300 μm binary particle systems, and the cluster rotation under a non-uniform rotating magnetic field.

The 2D rotating magnetic field can be programmed to regulate the collective motion of magnetic microparticles. The rotating magnetic field is expressed as

(1) $$B(t)=\left(B_{x}\sin(2\pi f_{x}t)\right)\cdot \hat{x}+\left(B_{y}\cos(2\pi f_{y}t+\Delta \varphi )\right)\cdot \hat{y}$$

where B(t) is the total magnetic field vector, B_x_ and B_y_ are the field amplitudes along the two orthogonal axes, t is time, *f*_x_ and *f*_y_ are the corresponding driving frequencies, and ∆*φ* denotes the phase difference between the magnetic field components along the x-directions and y-directions. When *f*_x_ = *f*_y_, Equation (1) results in a uniform rotation of the magnetic field and a circular shape of the total magnetic field vector B(t) in one period, which drives continuous particle spinning and induces collective rearrangement. While ∆*φ* = 90° and *f*_x_ and *f*_y_ are not identical, the superposition results in a non-uniform rotation of the magnetic field and an elliptical shape of B(t), resulting in pronounced spatiotemporal asymmetry in the magnetic torque and the induced hydrodynamic interactions.

When a rotating magnetic field is applied, the magnetic microparticles rotate synchronously with the external field within the synchronous regime, such that the particle rotation frequency follows the driving frequency. As the driving frequency approaches and exceeds the critical step-out frequency, this synchronous response gradually breaks down, as discussed in detail in Section S2 of the Supplementary Information. The rotation of each microparticle entrains the surrounding fluid, generating a rotational flow field whose tangential velocity decreases with increasing radial distance from the particle. The Reynolds number of this induced rotational flow field attains values of O(1)–O(10), indicating a finite Reynolds number regime. The flow field in this regime can be described the finite Reynolds number hydrodynamic interaction of spherical spinners reported by Shen et al. [27], which not only drives neighboring particles to move along the rotational direction, but also generates a hydrodynamic repulsive force. Meanwhile, under the actuation of the externally applied magnetic field, the motion of magnetic particles confined at the interface is also affected by magnetic dipole–dipole interaction forces, which can be expressed as

(2) $$F_{h}^{ij}(d)=C_{\mathrm{int}}\frac{\rho R_{i}^{4}R_{j}^{3}\omega_{j}^{2}}{d^{3}}$$

(3) $$F_{m}^{ij}\left(d\right)=-\frac{3\mu_{0}m_{j}m_{i}}{4\pi d^{4}}$$

(4) $$m_{i}=4\rho_{m}\pi R_{i}^{2}$$

where C_int_ is the numerical prefactor of the hydrodynamic interaction, which is modified relative to the unbounded case, ρ is the density of water, R_i_ and R_j_ are the radii of the i-th and j-th particles, respectively, ω_j_ is the angular velocity of the j-th particle, mi and m_j_ are the magnetic moments of the i-th and j-th particles, respectively, ρ_m_ is the magnetic moment per unit area, and d is the center-to-center distance between the two particles. We note that the finite Reynolds number hydrodynamic repulsion in Equation (2), adopted from the analysis of Wang et al. [16], is derived for isolated spherical spinners in an unbounded fluid. In the present experiments the particles are only partially submerged at the air–water interface, so the entrained flow is generated in the liquid sub-phase. Because the air–water interface can be approximated as a stress-free boundary, the numerical prefactor C_int_ of the hydrodynamic interaction is modified relative to the unbounded case, while its scaling and the repulsive sign of the radial force are preserved. Moreover, Equation (2) represents a pairwise interaction that is strictly valid in the dilute limit; at the moderate and high area fractions studied here, the overlap of the rotational flow fields of neighboring particles introduces additional many-body dissipation and positional fluctuation.

**Figure 2:**
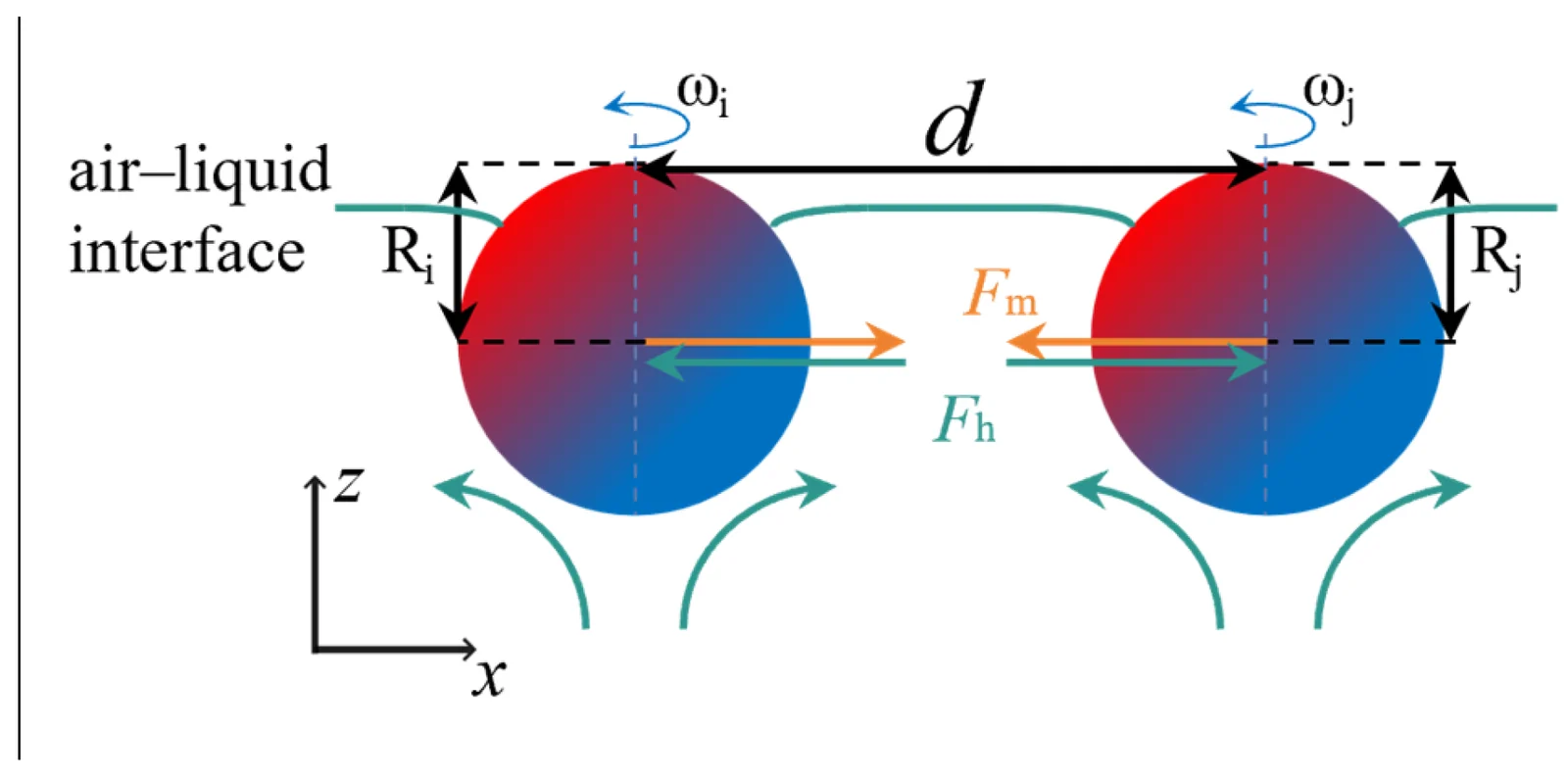
Force analysis of a pair of particles. The orange arrows represent the magnetic force, the green arrows represent the hydrodynamic force, and the black arrows represent the rotational direction. The blue-to-red color gradient represents the magnetic poles (N and S).

Because the particles reside at the air–water interface, we estimate the magnitude of the meniscus-induced lateral capillary attraction relative to the magnetic dipolar interaction. The interface deformation around a particle of radius R is set by the balance between the particle’s excess weight and the vertical component of the surface tension, which defines a capillary charge Q ≈ W/(2πγ), where W is the excess weight and γ the surface tension. The lateral capillary force between two identical particles separated by a distance d is Fcap ≈ 2πγqQ^2^K_1_, where q = 1/λc is the inverse capillary length (λc = (γ/ρg)1/2 ≈ 2.7 mm for water) and K_1_ is the modified Bessel function of the second kind. Using R = 150 μm, γ = 72 mN/m, and the excess weight of the present Ni-coated particles, we obtain Q~10^−8^–10^−7^ m and Fcap~10^−12^–10^−10^ N for adjacent particles, several orders of magnitude weaker than the magnetic dipolar force between neighboring particles (Fm~10^−8^–10^−6^ N). The small Bond number, Bo = ρgR^2^/γ ≈ 3 × 10^−3^ ≪ 1, further confirms that interfacial deformation is weak. Capillary attraction is therefore negligible compared with the magnetic and hydrodynamic interactions that govern the collective dynamics studied here. The competition between these two interactions is schematically illustrated in Figure 2. In this competitive system, each particle maintains self-rotation under the external field, while interparticle coupling through magnetic dipolar interactions and spin-induced hydrodynamic interactions drives the entire particle assembly to undergo orbital rotation around a common center.

By programming the magnetic field, we can modulate the dynamic balance between magnetic dipolar interactions and spin-induced hydrodynamic interactions, thereby inducing elongation, periodic approach–separation motion, and separation of particle assemblies.

## 3. Results

### 3.1. Collecitve Motion and Hexatic Order of Single-Component System

**Figure 3:**
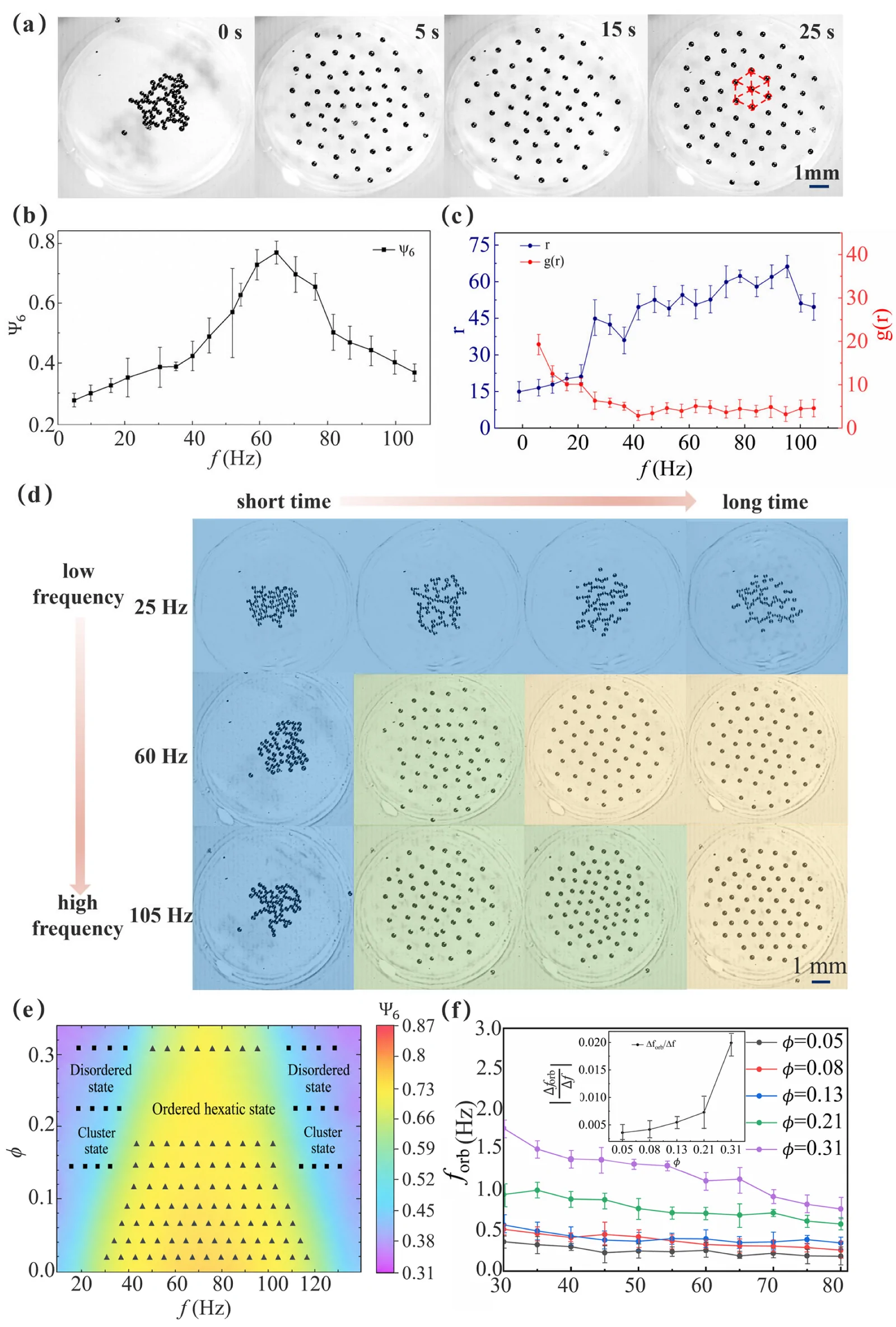
Structural evolution, frequency response, and phase behavior of single-component 300 μm magnetic microparticle assemblies driven by a rotating magnetic field. (**a**) Representative time-lapse images showing the evolution of magnetic microparticle assemblies from an initially aggregated state to a hexagonally ordered structure at an area fraction of *ϕ* = 0.13 and a driving frequency of *f* = 65 Hz. (**b**) Variation in the global hexatic order parameter Ψ_6_ with various driving frequencies *f* at *ϕ* = 0.13. (**c**) Variation in the characteristic interparticle distance r and the first peak value of the radial distribution function g(r) with the driving frequency *f* at *ϕ* = 0.13. (**d**) Time-resolved structural response of 300 μm magnetic microparticle assemblies at *ϕ* = 0.13 over a driving frequency range of *f* = 25 Hz, 60 Hz, and 105 Hz. The color scale ranges from cool (blue) to warm (orange) tones, indicating the degree of hexagonal ordering, with warm colors representing progressively better (higher) hexagonal order. (**e**) Steady-state *f*–*ϕ* phase diagram of single-component magnetic microparticle assemblies constructed based on the global hexatic order parameter Ψ_6,_ in which the system is classified into clustered, hexagonally ordered, and disordered states (the lowest area fraction was achieved with an assembly of seven particles). (**f**) Dependence of the collective orbital angular velocity *f*_orb_ on the driving frequency *f* at different area fractions *ϕ*. The inset shows the orbital response coefficient |Δ*f*_orb_/Δ*f*| as a function of *ϕ*. Error bars represent standard deviation calculated from *n* = 5 independent replicate experiments.

We first begin with the collective rotational motion of single-component magnetic microparticles and investigate the spontaneous emergence of ordered structures by tuning the rotation frequency and particle density (i.e., interparticle spacing). Figure 3a shows a representative example when *ϕ* = 0.13 and *f* = 65 Hz. The particle area fraction *ϕ* is expressed as

(5) $$\phi =\frac{N\pi R^{2}}{A}$$

where N is the number of particles, R is the particle radius, and A is the observation area. After the rotating magnetic field is applied, the initially compact particle cluster gradually expands and rearranges with time. The interparticle spacing becomes increasingly uniform, and the assembly finally evolves into a locally ordered hexatic structure, indicating that a dynamic balance is established between magnetic dipolar attraction and spin-induced hydrodynamic repulsion.

The degree of local hexagonal ordering is expressed as

(6) $$\psi_{6}(i)=\frac{1}{N_{i}}\sum_{j=1}^{N_{i}}e^{6i\theta_{ij}}$$

where *N_i_* is the number of nearest neighbors of particle *i*, and *θ_ij_* is the angle between the bond connecting particles *i* and *j* and a reference axis. The global order parameter is expressed as

(7) $$\Psi_{6}=\langle |\psi_{6}(i)|\rangle$$

To further characterize the spatial correlation of the microparticle assembly, the radial distribution function *g*(*r*) was expressed as

(8) $$g(r)=\frac{n(r,\Delta r)}{2\pi r\Delta r\rho_{N}}$$

where *n*(*r,*Δ*r*) is the particle number within the annular region between r and *r +* Δ*r*, and *ρ*_N_ is the number density of particles.

Figure 3b,c further quantify the effects of driving frequency on the degree of hexagonal ordering and the spatial distribution of the particles. As shown in Figure 3b, the global hexagonal order parameter Ψ6 exhibits a non-monotonic dependence on frequency, first increasing and then decreasing as the driving frequency increases. When the driving frequency reaches approximately *f* = 65–70 Hz, Ψ_6_ attains a maximum value of about 0.75, indicating that an optimal dynamic balance is established between magnetic dipolar attraction and spin-induced hydrodynamic repulsion. Under this condition, the microparticle assembly forms a relatively stable locally hexagonally ordered structure. As the magnetic field frequency further increases, the microparticles are unable to maintain synchronous rotation with the external magnetic field and gradually enter the step-out regime. This weakens the regular hydrodynamic coupling, thereby reducing the degree of hexagonal ordering. Meanwhile, Figure 3c shows that the driving frequency not only affects the hexagonal order parameter, but also significantly regulates the spatial correlation and characteristic interparticle spacing. As the frequency increases from the low-frequency regime, the main peak intensity of the radial distribution function g(r) increases markedly, indicating an enhanced probability of finding microparticles at the characteristic separation distance and a stronger local spatial correlation within the assembly. At the same time, the characteristic interparticle distance r gradually decreases and then approaches a stable value, suggesting that the particle spacing becomes more uniform and that the system evolves from a loose aggregate into a more ordered structure. This trend is consistent with the variation in Ψ_6_ shown in Figure 3b, further confirming that, within the intermediate frequency range, magnetic dipolar attraction and spin-induced hydrodynamic repulsion can reach a stable balance, thereby promoting the formation of hexagonal ordering.

We then systematically examined the hexagonally ordered structure (Figure 3d) by varying the driving frequency *f* and the particle area fraction *ϕ* obtained a phase diagram as shown in Figure 3e. In Figure 3d, we show snapshots of experimental videos to illustrate how the hexagonally ordered structures evolve under different rotational frequencies. We observe that at a low driving frequency (*f* = 25 Hz), the weak spin-induced flow field is insufficient to overcome the magnetic dipolar attraction and the constraint of the initial aggregates, and the particles therefore remain locally clustered. At an intermediate frequency (*f* = 60 Hz), the enhanced hydrodynamic interaction drives the initially compact cluster to expand and rearrange into a stable hexagonally ordered structure. When the frequency is further increased to 105 Hz, strong overlap and shear between neighboring rotational flow fields increase viscous dissipation and positional fluctuations, thereby reducing the hexatic order. We use a colorbar shown in Figure 3e to represent the global hexatic order parameter “Ψ_6_”. which displays high hexatic order in white and low order in blue. The phase diagram demonstrates that the highest hexatic order parameter appears in the region of intermediate driving frequency (*f* = 60–80 Hz) and low particle area fraction *ϕ* close to zero. This result indicates a significant effect of the hydrodynamic interaction among the rotational microparticles. As the driving frequency increases from low to intermediate values, the enhanced spin-induced flow strengthens the hydrodynamic coupling between neighboring particles, promoting cluster expansion, particle rearrangement, and the formation of a nearest-neighbor structure with sixfold symmetry. At higher rotational speeds, the oppositely directed rotational flows induced by neighboring particles enhance viscous dissipation and consequently disturb the ordered structure. With rising rotational speed, the rotational Reynolds number ReΩ=2πfρR2/μ increases from approximately 1.41 at 10 Hz to 14.14 at 100 Hz, R = 150 μm and *μ* = 10^−3^ pa·s. Under finite ReΩ, the growing inertial contribution modifies the flow field surrounding each spinning microsphere and introduces inertial hydrodynamic effects, which compete with viscous coupling and perturb the equilibrium particle configuration. Similarly, increasing particle area fraction *ϕ* reduces the interparticle spacing and thereby strengthens hydrodynamic interaction. Therefore, we observe that the hexatic order parameter decreases with the increasing particle area fraction within the investigated range. At a relatively low particle fraction, the particles retain sufficient space for lateral migration and structural rearrangement. The spin-induced hydrodynamic interaction can promote coordinated motion between neighboring particles without introducing excessive many-body flow interference, allowing local positional deviations and coordination defects to gradually relax and thereby facilitating the formation of a hexagonally ordered structure. As the particle fraction increases, the reduced interparticle spacing leads to a stronger overlap of the rotational flow fields generated by neighboring particles. The relative opposition of local flow velocities enhances viscous dissipation and velocity fluctuations, while geometric crowding restricts particle outward migration and forms the hexagonally ordered structure in the middle. Consequently, although the hydrodynamic interaction becomes stronger at higher particle fractions, the accompanying many-body flow interference and limited rearrangement space ultimately reduce the hexatic order. At high particle fractions, the increased viscous resistance and flow dissipation cause more input energy to be dissipated through local fluid shear, thereby suppressing effective particle rearrangement and further decreasing the hexatic order. We notice that the highest hexatic order parameter corresponds to an interparticle spacing of approximately 707 μm, which is approximately twice the particle diameter.

In addition to local structural ordering, the rotating magnetic field also induces a collective orbital motion of the microparticle assembly around the center of the system aside from the rotation of each individual microparticle. Figure 3f shows that the orbital frequency forb depends on the particle area fraction *ϕ*. At the same driving frequency, a higher *ϕ* leads to a larger forb. This is because an increase in particle number density reduces the interparticle spacing, thereby enhancing both many-body hydrodynamic coupling and magnetic dipolar interactions. As a result, the local flow fields induced by individual spinning particles are more readily superimposed at the cluster scale, generating cooperative rotation and increasing the overall orbital speed. On the other hand, as the driving frequency increases from 30 Hz to 80 Hz, forb decreases for all values of *ϕ*. This result indicates that the collective orbital motion does not simply increase with the single-particle spin frequency, but is jointly limited by particle synchrony and the structural stability of the assembly. At higher frequencies, the periodic disturbance of the flow field around the particles is enhanced and viscous dissipation increases, which instead leads to a decrease in the orbital frequency. This is because, as the driving frequency increases, the periodic disturbance of the flow field around the particles is enhanced and viscous dissipation increases, leading instead to a decrease in the orbital frequency. The inset shows that |Δ*f*_orb_/Δ*f*| increases with *ϕ*, indicating that an increase in the particle spinning frequency weakens the effective orbital response at the cluster scale, and that this negative response becomes more pronounced in high-density systems.

### 3.2. Phase Separation of Binary Microparticle Assemblies

**Figure 4:**
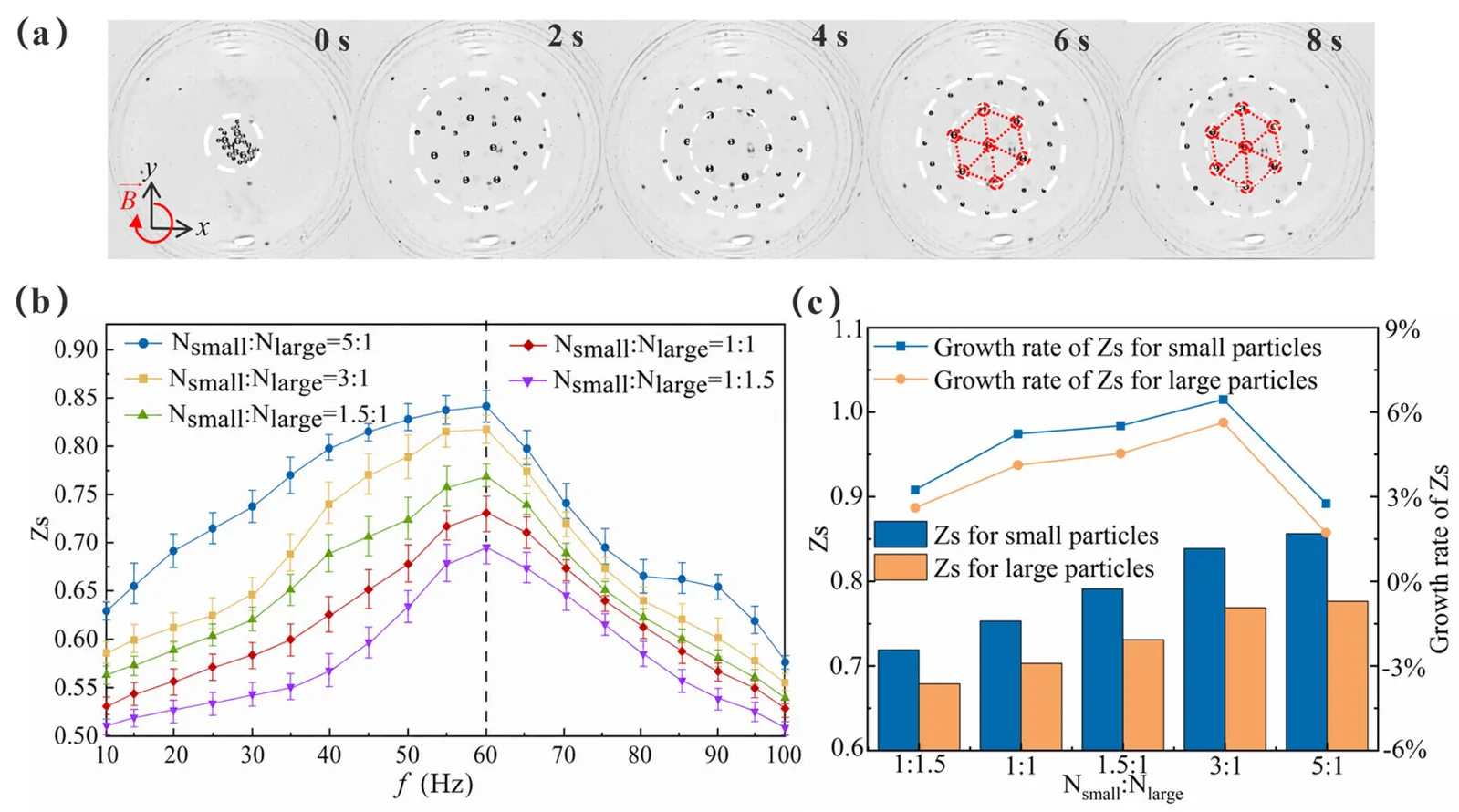
Phase separation of binary magnetic microparticle assemblies. (**a**) Time-lapse images of a binary particle assembly composed of 200 μm and 300 μm magnetic microparticles at *f* = 60 Hz, showing the transition from a mixed state to a separated state. (**b**) Separation parameter Zs as a function of driving frequency *f* under different number ratios of small to large particles, N_small_:N_large_. (**c**) The Zs values and corresponding growth rates of small and large particles at different particle number ratios. Error bars represent standard deviation calculated from n = 5 independent replicate experiments.

It is natural to ask how the assembly structure changes if the microparticle system is changed to a binary system of two particle sizes. In the experiment, the diameter of the larger microparticle is 300 μm, and that of the smaller particle is 200 μm. The number ratio of small to large microparticles N_small_:N_large_ is varied from 1:1.5 to 5:1. After applying the rotating magnetic field, we observe a phase separation phenomenon of the binary microparticle assembly (as shown in Figure 4a, *f* = 60 Hz). As the microparticles disperse from the aggregated state, the smaller microparticles disperse faster than the larger ones, leading to a phase-separated structure in which large microparticles form a hexagonally ordered structure in the interior, whereas small microparticles encapsulate the outer layer. The phase separation process takes less than 10 s in the case shown in Figure 4a, where the *Re*_Ω_ = 8.48, calculated based on the rotational flow of the 300 μm particles, when *f* = 60 Hz, indicating the significant hydrodynamic effect of the finite Re number flows. Stronger repulsive flow fields develop around larger microparticles, whereas smaller particles are more susceptible to destabilization and are repelled toward the outer layer. Thus, the phase-separated structure arises because the hydrodynamic coupling among different particles drives a rearrangement into a more stable configuration.

We introduce a separation parameter Zs to quantify the phase separation state of the binary system. The separation parameter Zs is defined as [22]

(9) $$Zs=\frac{1}{N}\sum_{i=1}^{N}\frac{S_{i}}{n_{i}}$$

To minimize the influence of boundary effects on the statistical evaluation of the degree of separation, particles in the outermost layer of the assembly were excluded from the calculation of Zs, and the remaining particles were considered. Here, N is the number of particles included in the calculation, S_i_ is the number of particles of the same size as particle i within its local neighborhood, and $n_{i}$ is the total number of nearest neighbors of particle i. The neighboring relationships are determined using Voronoi tessellation, where two particles sharing a common Voronoi edge are considered neighbors [22]. A larger Zs indicates stronger local enrichment of particles of the same size and thus a higher degree of size segregation.

In Figure 4b, we show two-stage variations in Zs with the driving frequency *f* under different particle number ratios N_small_:N_large_. For all particle number ratios, Zs first increases and then decreases with increasing frequency. At low frequencies, the weak spin-induced hydrodynamic interactions are insufficient to disrupt the mixed clusters. As the frequency increases from 40 to 60 Hz, enhanced hydrodynamic interactions promote particle migration and rearrangement, leading to an increase in Zs. At higher frequencies, increased phase lag and unsteady flow disturbances cause local remixing, resulting in a gradual decrease in Zs.

Intriguingly, all the peak values of Zs are located at the same driving frequency *f* = 60 Hz, suggesting that a favorable dynamic balance between magnetic dipolar interactions and hydrodynamic interactions is established at this frequency. The higher the particle number ratio N_small_:N_large_ is, the larger the value of Zs is. This indicates that increasing the fraction of small particles promotes their local enrichment and enhances the overall segregation of the binary system.

Furthermore, we show the segregation parameters and growth rates (defined as the variation rate of Zs with the particle number ratio) of the small and large particles in Figure 4c. The small particles exhibit higher Zs values and greater sensitivity to the particle number ratio, indicating that the overall segregation is mainly governed by the rearrangement of small particles. The ratio N_small_:N_large_ = 3:1 does not yield the highest final segregation degree but corresponds to the fastest increase in the segregation of small particles (i.e., highest increasing rate of Zs). This nonlinear behavior results from the combined effects of particle number ratio, magnetic response differences, geometric constraints, and hydrodynamic coupling.

### 3.3. Non-Circular Pattern Through Programmed Rotating Magnetic Field Waveforms

**Figure 5:**
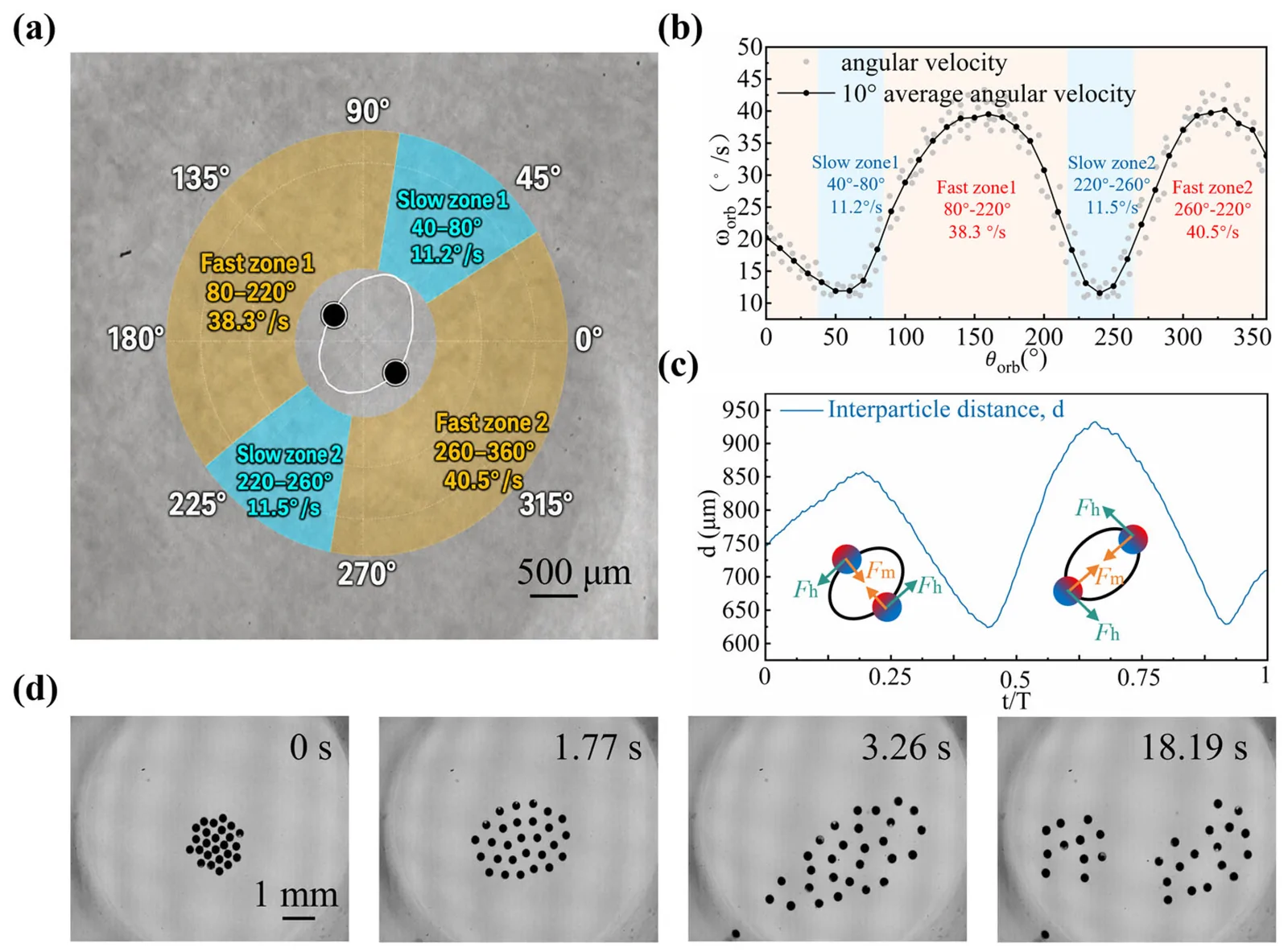
Separation behavior of particle assemblies under a programmed magnetic field of *f*_x_:*f*_y_ = 1:3. (**a**) Orbital trajectory of a two-particle system and the corresponding fast and slow angular zones during one orbital cycle. (**b**) Orbital angular velocity ω_orb_ as a function of orbital direction angle *θ*_orb_. Gray dots and the black curve represent the frame-resolved angular velocity and the 10–binned average angular velocity, respectively. (**c**) Temporal evolution of the interparticle distance *d*. The orange arrows represent the magnetic force, the green arrows represent the hydrodynamic force. (**d**) Time-lapse images of a particle assembly evolving from an aggregated state to a non-circular shape, and finally separated into two subclusters, under a programmed magnetic field of *f*_x_:*f*_y_ = 1:3.

We can tune the frequencies of *f*_x_ and *f*_y_ separately (see Equation (1) and Figure 1c) to vary the superposition of the rotating magnetic field. When *f*_x_ and *f*_y_ are not identical, a non-uniform rotation of the magnetic field is obtained. Such a non-uniform rotating magnetic field leads to more diverse collective structures. Here, we set *f*_x_:*f*_y_ = 1:3 to perform experiments, and the magnetic field amplitudes are set as B_x_ = 3B_y_ = 1.03 mT. As shown in Figure 5a, one orbital cycle can be divided into fast and slow zones according to the orbital velocity of the particle pair in different directions. The angular intervals of 40–80° and 220–260° correspond to the slow zones, whereas 80–220° and 260–360° correspond to the fast zones. The distributions of the two fast and two slow zones demonstrate the pronounced dependence of particle rotation under this 1:3 superpositioned magnetic field.

To quantitatively characterize this non-circular rotation pattern of a pair of microparticles, Figure 5b shows the variation in the orbital angular velocity ω_orb_ with the orbital direction angle *θ*_orb_. Although the frame-resolved experimental data exhibit fluctuations, the direction angle-binned average curve displays reproducible peaks and valleys. The peaks correspond to the fast zones, whereas the valleys correspond to the slow zones. This result confirms that the acceleration and deceleration of the rotating microparticle pair are consistently regulated by the applied magnetic field waveform.

To understand how the non-uniform orbital motion produces an elliptical trajectory, we further examine the evolution of the interparticle distance in a two-particle system composed of particles of identical size. As shown in Figure 5c, the interparticle distance d varies periodically. Due to the viscous response of the surrounding fluid, the radial displacement of the particles lags behind the field-driven rotational motion. Consequently, after the particles leave a fast zone, the hydrodynamic effect generated in the preceding stage continues to drive them outward, so that the interparticle distance further increases before reaching the maximum value. As the particles move apart, the spin-induced hydrodynamic coupling between them gradually weakens and the tangential driving effect declines. When the hydrodynamic interaction becomes sufficiently weak, magnetic dipolar attraction drives the particles to approach each other. The reduced interparticle distance strengthens the hydrodynamic coupling again. The combined effects of phase lag and distance-dependent hydrodynamic coupling led to the periodic variation in interparticle distance d. These effects also produce direction-dependent radial displacements, eventually forming an approximately elliptical trajectory for the particle pair.

The above mechanism of the rotating microparticle pair can be further extended to a multiparticle system to regulate its non-circular rotating pattern. As shown in Figure 5d, the multiparticle system initially forms a compact aggregate with an approximately circular outline. After application of the modulated magnetic field, spin-induced hydrodynamic interactions drive the aggregate to expand outward, accompanied by an increase in interparticle spacing. With increasing driving time, the hydrodynamic effect progressively breaks the initial aggregation and induces more pronounced radial migration of the particles along a preferred direction, causing the system to gradually evolve from a nearly circular configuration into an elliptical structure. At longer times, inhomogeneous particle distribution or random internal perturbations may produce an imbalance in the dynamic response of different regions, leading to localized necking and ultimately causing the aggregate to split into two subclusters. The experiment provides insight into the non-circular deformation of the rotating multiparticle assembly that is seldom reported in existing experiments. Interestingly, the superposition of the 2D magnetic field recalls the complex patterns formed by the interaction of two perpendicular harmonic oscillations [28]. These results demonstrate that the collective configuration can be effectively regulated by programming the temporal waveform of the external magnetic field.

Although this study focuses on the representative case of *f*_x_:*f*_y_ = 1:3 with Δφ = 0°, the programmable magnetic field framework provides additional degrees of freedom for regulating collective behaviors. Changing the frequency ratio *f*_x_:*f*_y_ modifies the temporal variation in the magnetic field rotation velocity, which can generate different types of non-circular trajectories and asymmetric collective motions. For example, frequency ratios closer to unity are expected to produce weaker directional asymmetry, whereas larger frequency mismatches can introduce more pronounced variations in the angular velocity and induce higher-order periodic deformation patterns. In addition, varying the phase difference Δφ between the orthogonal magnetic field components changes the symmetry and orientation of the resulting trajectories, providing an additional parameter for controlling cluster deformation and separation directions. Therefore, the combination of frequency ratio and phase difference offers a versatile strategy for manipulating collective structures beyond the specific condition investigated here. Such open-loop magnetic field programming provides a foundation for further developing adaptive control strategies.

From a control perspective, closed-loop strategies for magnetically actuated microrobots have been extensively developed, including feedback control of self-organized spinning micro-disks [29], adaptive cascade navigation of magnetic microrobots in unstructured dynamic environments [30], and path planning and tracking of underactuated two-microrobot systems [31]. The programmable-field framework presented here can be naturally integrated with such closed-loop regulations. For example, by monitoring the instantaneous hexatic order parameter, the segregation parameter, or the swarm centroid in real time, the driving frequency, field amplitude, or frequency ratio could be adaptively adjusted to maintain the assembly near a target state, to counteract drift of the phase-separated structure, or to preserve a desired interparticle spacing. The non-monotonic frequency dependence of Ψ_6_ and Zs identified in this work (Figure 3 and Figure 4) offers a quantitative basis for selecting and updating the operating point of such adaptive regulators, and the programmable waveforms provide the actuation bandwidth required for closed-loop reconfiguration of the swarm spatial distribution, phase separation dynamics, and interparticle interactions.

## 4. Discussion

Existing studies on rotating active particles largely ignore air–liquid interfacial confinement and either adopt dilute bulk suspensions or fix particle packing density, leaving the coupled influence of driving frequency and area fraction on magnetic–hydrodynamic competition underexplained. Contrary to conventional hypotheses that magnetic moment disparity dominates binary particle segregation, our experiments verify size-dependent hydrodynamic repulsion as the core driver of phase separation; meanwhile, programmable Lissajous-type magnetic fields break the widely assumed circular orbital symmetry via fluid viscous lag, providing new insights into anisotropic collective reorganization unique to 2D interfacial systems.

All observed mechanisms are limited to rotating microparticles located at air–liquid interfaces, where interfacial tension might influence local flow fields. Future work will introduce chiral spinning units, extend the system to 3D confinement, and test richer magnetic frequency ratios, so as to generalize the competing interaction framework and advance the design of reconfigurable microrobots swarm across various fluid interfaces.

## 5. Conclusions

This work systematically uncovers the collective self-organization of magnetically driven rotating microparticles located at air–liquid interfaces, which can be explained by the competition between magnetic dipolar attraction and finite Reynolds number hydrodynamic repulsion. Our experiments show rich phenomena of the collective motion of the rotating active microparticles, including the formation of hexagonally ordered structures, the phase separation of binary microparticles, and the uncommon non-circular pattern generated by programmable magnetic fields. For single-component assemblies, hexagonally ordered structures only emerge at intermediate driving frequencies where the two forces reach dynamic equilibrium, while low frequencies keep particles clustered and excessive high frequencies destroy long-range order via enhanced viscous dissipation. The constructed frequency–area fraction phase diagram supplements prior fixed-density studies, and the cluster orbital frequency positively correlates with particle area fraction yet declines with driving frequency, a rarely quantified phenomenon in homogeneous spinner systems. Binary particle mixtures exhibit hydrodynamic-induced core–shell size segregation with the segregation parameter Zs peaking universally at 60 Hz, and lightweight small particles dominate rearrangement to realize non-equilibrium particle sorting different from thermal separation of passive colloids. By applying Lissajous-type magnetic fields with mismatched orthogonal frequencies (*f*_x_:*f*_y_ = 1:3), fluid viscous lag generates alternating fast and slow orbital zones and periodic fluctuations in interparticle distance, enabling controllable elliptical deformation and splitting of particle clusters, which provides a novel programmable manipulation strategy distinct from conventional uniform rotating magnetic actuation. The systematical results of the controlled collective motion shown in this study shed light on better manipulation in the applications of swimming microrobot swarm. Future work will be expanded to wider parameter spaces including chiral microparticles and various magnetic fields.

## Data Availability

The original contributions presented in this study are included in the article and Supplementary Material. Further inquiries can be directed to the corresponding authors.

## Supplementary Materials

The following supporting information can be downloaded at https://www.mdpi.com/article/10.3390/mi17091068/s1, Figure S1: Magnetic field generated by the orthogonal Helmholtz coils and its spatial distribution characteristics; Figure S2: Force analysis and rotational dynamics of a magnetic particle under a magnetic field.
