# Supplementary material for: Collective Motion and Programmable Self-Organization of Rotating Active Particles Manipulated by Magnetic Fields
Source: Micromachines (Basel). 2026 Sep 9;17(9):1068. doi: 10.3390/mi17091068 (PMC13608911; doi:10.3390/mi17091068)
Supplement: Supplementary file 1 [file micromachines-17-01068-s001.zip › micromachines-4532358-supplementary.pdf]

Collective Motion and Programmable Self-Organization of Rotating Active Particles Manipulated by Magnetic Fields

Kailai Wang[1,2], Jiawei Li[3], Leilei Wang*[,1] Shishuang Zhang[2], Xu Zheng[1], Qilong Zheng[3], and Haihang Cui[*,2]

1 State key laboratory of nonlinear mechanics, Institute of Mechanics, Chinese Academy of Sciences, Beijing,100190, People’s Republic of China.
2 School of Building Services Science and Engineering, Xi’an University of Architecture and Technology, Xi’an, 710055, People’s Republic of China.
3 Xi’an Modern Chemistry Research Institute, Xi’an 710065, China.

* Correspondence: wangleilei@imech.ac.cn; cuihaihang@xauat.edu.cn; Author to whom correspondence should be addressed

* Corresponding Authors:

Leilei Wang: wangleilei@imech.ac.cn, https://orcid.org/ 0000-0002-0379-8973;

Haihang Cui: cuihaihang@xauat.edu.cn

S1: Magnetic field uniformity

Programmable electrical signals generated by a signal generator were amplified and independently applied to two orthogonal pairs of Helmholtz coils to generate a two-dimensional magnetic field in the horizontal plane. The configuration and magnetic field model of the orthogonal Helmholtz–coil system are shown in Fig. S1(a). For an ideal Helmholtz–coil pair, the magnetic field amplitude at the geometric center is given by

$$B_0 = \left(\frac{4}{5}\right)^{3/2} \frac{\mu_0 NI}{R_c} \qquad \text{(S1)}$$

where μ_0 is the vacuum permeability, N is the number of turns of each coil, I is the amplitude of the driving current, and R_c is the radius of the Helmholtz coil. The magnetic field amplitudes used in the experiments were further calibrated experimentally at the center of the coil system. The spatial distribution of the magnetic field was further evaluated using finite-element simulations in COMSOL Multiphysics, following established numerical modeling approaches for orthogonal Helmholtz–coil systems [26]. The simulation geometry was consistent with the experimental setup, with a driving current of 7 A with frequency of 60Hz, 100 turns per coil, and a coil separation of 4 cm, The programmable rotating magnetic field, generated by the superposition of sine and cosine waves along the x and y axes, had a magnitude of approximately 1.0 mT at the center. As shown in Fig. S1(b), the magnetic field is nearly uniformly distributed within the central region (black circle of 1.0 cm diameter) of the coil system. The arrows indicate the magnetic field direction, and the nearly parallel field lines further demonstrate the spatial uniformity of the field. To quantitatively evaluate the field uniformity, the magnetic field magnitude in the central region was further analyzed, as shown in Fig. S1(c), with the center of the uniform magnetic field region defined as the coordinate origin. The magnetic field magnitude exhibits only small spatial fluctuations around the central value, and the field uniformity within the effective manipulation region exceeds 90%. These results confirm that spatial variations in the applied magnetic field are sufficiently small and can be neglected over

the experimental observation area.

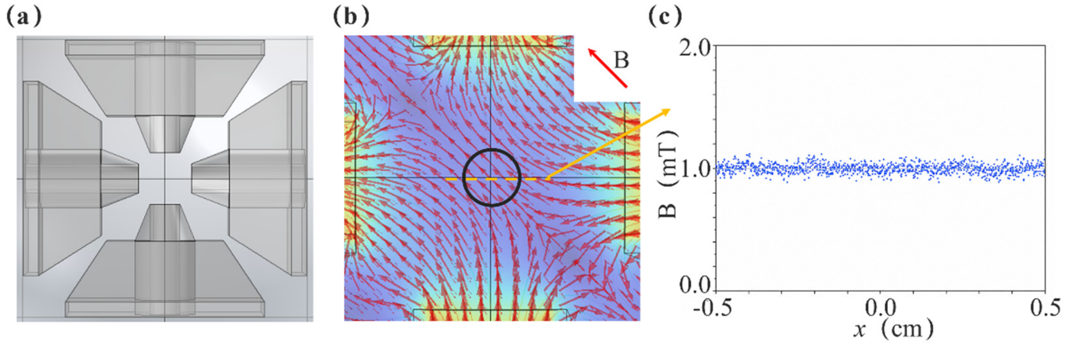

Figure S1. Magnetic field generated by the orthogonal Helmholtz coils and its spatial distribution characteristics. (a) Magnetic field model of the orthogonal Helmholtz coils; (b) spatial distribution of the magnetic field, where the black circle (diameter of 1.0 cm) denotes the uniform magnetic field region and a sampling plane is defined at the center of this region to extract the magnetic field magnitude; (c) magnetic field uniformity in the central region of the Helmholtz coils obtained from the sampling plane defined in (b), with the center of the uniform magnetic field region taken as the coordinate origin.

S2: Characterization of the critical step-out frequency

Synchronous rotation of a particle requires that the magnetic torque, T_m = mB·sinθ (with θ the lag angle between the particle's magnetic moment and the local field direction), balance the viscous drag torque $T_v \approx c\mu R^3\omega$, where R is the particle radius, c is a geometry-dependent prefactor (c = 8π for a sphere in an unbounded fluid; a smaller value applies to a partially submerged particle at a free interface) and ω is angular velocity. The schematic diagram of forces acting on a magnetic particle under a magnetic field is shown in Fig. 2(a). Because the lag angle grows with the driving frequency, the particle can no longer follow the field once θ reaches 90°, which defines the step-out frequency f_c=mB/(2πcμR^3). To quantitatively characterize the synchronization limit of the particles, we measured the single-particle spinning frequency f_p as a function of the applied magnetic field frequency f_m, as shown in Fig. S2(b). We identified the onset of the step-out regime for the particle with the diameter of 300 μm particles at $f_c \approx$ 120 Hz at the field amplitude used in the main experiments; consistent with the torque-balance prediction. When the applied magnetic field

frequency further exceeds f_c, the magnetic torque can no longer sustain synchronous rotation of the particle with the magnetic field. Consequently, the particle spinning frequency f_p gradually deviates from the field frequency f_m, indicating that the particle enters the step-out regime.

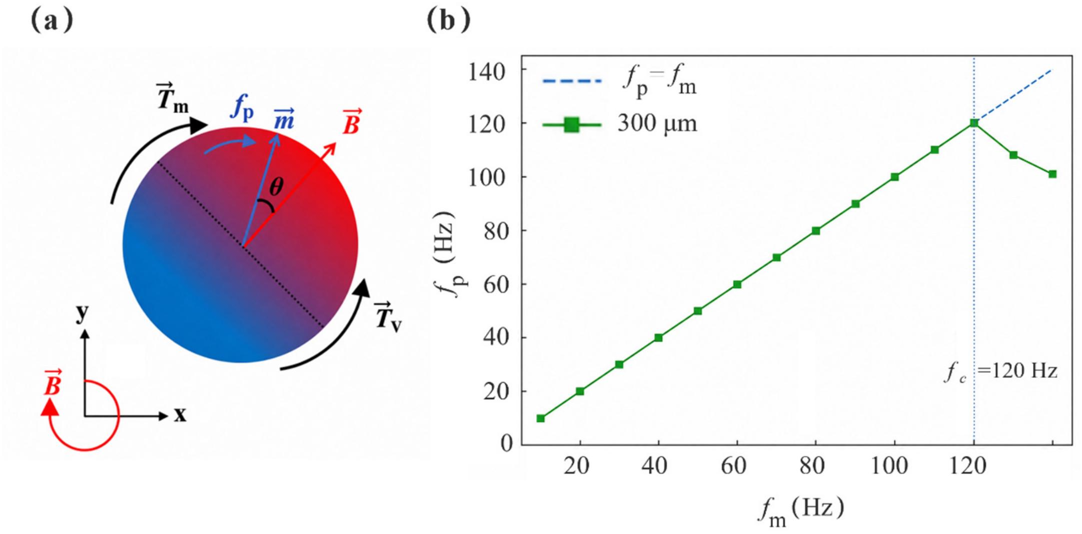

Figure S2. Force analysis and rotational dynamics of a magnetic particle under a magnetic field (a) The schematic diagram of forces acting on a magnetic particle under a magnetic field. (b) Variation in particle rotation frequency with magnetic field frequency.

References

[26] Shen, H.; Cai, S.; Wang, Z.; Yuan, Z.; Yu, H.; Yang, W. A Programmable Inchworm-Inspired Soft Robot Powered by a Rotating Magnetic Field. *Journal of Bionic Engineering* **2023**, 20, 506–514.
